# A new loach species of the genus *Paranemachilus* (Cypriniformes, Nemacheilidae) from Guangxi, China

**DOI:** 10.3897/zookeys.1269.173359

**Published:** 2026-02-13

**Authors:** Cai-Huan Mo, Jia-Jun Zhou, Min-Jie Deng, Zhuo-Cong Wang, Li-Na Du, Zheng-Quan Nong

**Affiliations:** 1 Key Laboratory of Ecology of Rare and Endangered-Species and Environmental Protection, Guangxi-Normal University, Ministry of Education, Guilin, Guangxi-541004, China Guangxi Key Laboratory of Rare and Endangered Animal Ecology, College of Life Sciences, Guangxi Normal University Guilin China; 2 Guangxi Key Laboratory of Rare and Endangered Animal Ecology, College of Life Sciences, Guangxi Normal University, Guilin, Guangxi 541004, China Key Laboratory of Ecology of Rare and Endangered-Species and Environmental Protection, Guangxi-Normal University, Ministry of Education Guilin China; 3 Zhejiang Forest Survey Planning and Design Company Limited, Hangzhou 310020, China Zhejiang Forest Resource Monitoring Center Hangzhou China; 4 Zhejiang Forest Resource Monitoring Center, Hangzhou, Zhejiang 310020, China Zhejiang Forest Survey Planning and Design Company Limited Hangzhou China; 5 Changbaishan Academy of Science, Erdaobaihe, Jilin 133613, China Changbaishan Academy of Science Erdaobaihe China; 6 Administration Center of Guangxi Nonggang National Nature Reserve, Longzhou, China Administration Center of Guangxi Nonggang National Nature Reserve Longzhou China

**Keywords:** Cryptic species, karst region, postcleithrum

## Abstract

A new species of *Paranemachilus*, *Paranemachilus nonggangensis***sp. nov**. is described from the Zuojiang river basin, a tributary of the Pearl River system. The new species can be differentiated from other members of the genus by morphological characters and molecular evidence. In morphology, the new species can be distinguished from other congeneric species by having scaled cheeks, the postcleithrum present, 12 or 13 branched pectoral fin rays, six branched pelvic fin rays, 11–15+3 infraorbital canal pores, and 6–10 supraorbital canal pores. A Bayesian inference phylogenetic analysis based on the mitochondrial Cyt *b* gene strongly supports the validity of *P. nonggangensis***sp. nov**.; the uncorrected *p*-distance is 5.18–7.67%.

## Introduction

The genus *Paranemachilus* Zhu, 1983 (family Nemacheilidae) comprises a taxonomically dynamic group of cave-adapted stone loaches endemic exclusively to the karst region of Guangxi Zhuang Autonomous Region, China. *Paranemachilus* was erected with *Paranemachilus genilepis* Zhu, 1983 as the type species and originally diagnosed by the following key morphological traits: cheeks scaled, postcleithrum present, bony capsule of the open-type, and posterior chamber well developed (Zhu 1983). For nearly three decades, this genus remained monotypic until the description of *P. pingguoensis* Gan, 2013, collected from Pingguo County, Guangxi, which uniquely lacks cheek scaleless and thus challenges the earlier diagnostic emphasis on scaled cheeks (Lan et al. 2013). Taxonomic revisions accelerated thereafter; Du et al. (2021) transferred *Yunnanilus jinxiensis* Zhu, Du, Chen & Yang, 2009 to *Paranemachilus* based on both cheek-scaling patterns and molecular evidence. Subsequent studies expanded the genus further, with descriptions of *P. chongzuo* Du, Xu, Luo & Zhou, 2023 from Chongzuo County (Du et al. 2023), followed by *P. luegvetensis* Mo, Yang, Li & Du, 2025 and *P. liui* Mo, Yang, Li & Du, 2025 from Wuming County, Nanning City, and Liuzhou City, Guangxi, respectively (Mo et al. 2024).

A critical taxonomic debate centred on the genus *Heminoemacheilus*, which was erected by Zhu and Cao (1987) based on the absence of a postcleithrum and the lack of cheek scales. While Bănărescu and Nalbant (1995) argued that postclethrum presence or absence was an interspecific rather than intergeneric trait, *Heminoemacheilus* continued to be recognized as a valid genus in subsequent literature (Prokofiev 2010; Kottelat 2012; Du et al. 2021). This classification was ultimately revised by Luo et al. (2023), who synonymized *Heminoemacheilus* with *Paranemachilus* using molecular evidence. Consequently, *Heminoemacheilus zhengbaoshani* Zhu & Cao, 1987 was reassigned to *Paranemachilus* as *P. zhengbaoshani* (Zhu & Cao, 1987), while other former *Heminoemacheilus* species were transferred to two newly established genera: *Guinemachilus* Du et al., 2023 and *Karstsinnectes* Zhou, Luo, Wang, Zhou & Xiao, 2023 (Du et al. 2023; Luo et al. 2023).

Consequently, the modern classification of *Paranemachilus* now places greater emphasis on nostril structure and molecular data, moving beyond earlier diagnostic traits like scaled cheeks and the presence of a postcleithrum. Today, the genus encompasses seven valid species: *P. chongzuo*, *P. genilepis*, *P. jinxiensis* (Zhu, Du, Chen & Yang, 2009), *P. liui*, *P. luegvetensis*, *P. pingguoensis*, and *P. zhengbaoshani*, each adapted to the unique hydrology of Guangxi’s karst systems. Morphologically, the genus is genetically and phenotypically diagnosed by a suite of consistent traits: a robust body, whole body covered by scales (with intraspecific variation in cheek scaling, either scaled or scaleless), suborbital spine absent, anterior and posterior nostril adjacent (with the anterior nostril base being tube-shaped and its tip bearing a barbel-like elongation), lateral line incomplete, postcleithrum variably present or absent, and anterior air bladder enclosed in a bony capsule (posteriorly membranous), paired with a posterior air bladder free in the abdominal cavity (Zhu 1983; Du et al. 2023).

In October 2024, 15 specimens were collected from the Administration Centre of Guangxi Nonggang National Nature Reserve, Chongzuo City, which is within the Zuojiang river drainage, Xijiang river system in Guangxi Zhuang Autonomous Region, China. A detailed morphological examination combined with a mitochondrial DNA analysis indicated that these specimens represent a previously undocumented species of *Paranemachilus*, which is formally described herein.

## Materials and methods

### Specimen collection and preservation

Field sampling procedures adhered to the Guide to Collection, Preservation, Identification, and Information Share of Animal Specimens (Xue 2010) and the Implementation Rules of Fisheries Law of the People’s Republic of China. All protocols concerning animal care and use complied with the regulations set forth by the Chinese Laboratory of Animal Welfare and Ethics guidelines (GB/T 35892-2018). Specimens were euthanized immediately upon collection by an overdose of anesthetic clove oil to minimize suffering. For molecular analyses, five specimens were preserved in 99% ethanol, stored at −20 °C, and deposited in the Guangxi Normal University (**GXNU**). An additional 10 specimens were fixed in 10% formalin for morphological examination, maintained in the Kunming Natural History Museum of Zoology, Kunming Institute of Zoology (**KIZ**), Chinese Academy of Sciences (CAS). Five specimens were scanned with an energy beam of 80 kV and a flux of 80 × μA using 360° rotation, then reconstructed into a 4,096 × 4,096 matrix of 1,536 slices. The final CT reconstructed skull images were exported with a minimum resolution of 8.9 μm. Skull images were exported from the virtual 3D model reconstruction using Volume Graphics Studio v. 3.4.0.

### Phylogenetic analysis

Genomic DNA was extracted from ethanol-preserved fin tissues using a commercial DNA extraction kit (Sangon Biotech (Shanghai) Co., Ltd, China). The mitochondrial cytochrome *b* gene (Cyt *b*) gene was amplified using the primer pair F14724 (5'-GACTTGAAAAACCACCGTTG-3') and R15915 (5'-ctccgatctccggattacaagac-3') following the protocols of Xiao et al. (2001) and sequenced, with the resulting data submitted to GenBank (accession nos. PX437380 to PX437383). To infer the phylogenetic placement of *Paranemachilus nonggangensis* sp. nov., 26 Cyt *b* sequences representing species of Nemacheilidae were retrieved from GenBank. Sequence alignment was performed in MEGA v. 11.0 (Tamura et al. 2021) using the MUSCLE algorithm (Edgar 2004) with default parameters. Phylogenetic relationships were reconstructed using both maximum-likelihood (ML) and Bayesian inference (BI) approaches, implemented through the CIPRES Science Gateway (Miller et al. 2010). ML analysis employed a rapid bootstrapping strategy with 1,000 bootstrap iterations. BI analysis was conducted in MrBayes v. 3.2.7a (Ronquist et al. 2012) with two independent runs of four Markov chains initiated from a random tree. The chains were run for five million generations and sampled every 100 generations, with the first 25% of sampled trees discarded as burn-in. The optimal nucleotide substitution model for Cty *b* was determined under the AICc criterion, using PartionFinder v. 2.1.1, identified as GTR+I+G. The remaining trees were used to create a consensus tree and estimate Bayesian posterior probabilities (BPPs). The constructed phylogenetic trees were visualized and edited in FigTree v. 1.4.4 (Rambaut 2009).

### Morphological examination

Meristic counts, morphometric measurements, and cephalic lateral line system features were assessed following the protocols outlined by Kottelat (1990). All measurements were taken from the left side of each specimen using digital vernier callipers to the nearest 0.1 mm. All measurements were recorded, with data processed and analyzed in Microsoft Excel. Abbreviations: **SL**, standard length; **TL**, total length; and **HL**, lateral head length.

## Results

### Genetic evidence from phylogenetic analysis

**Figure 1.**
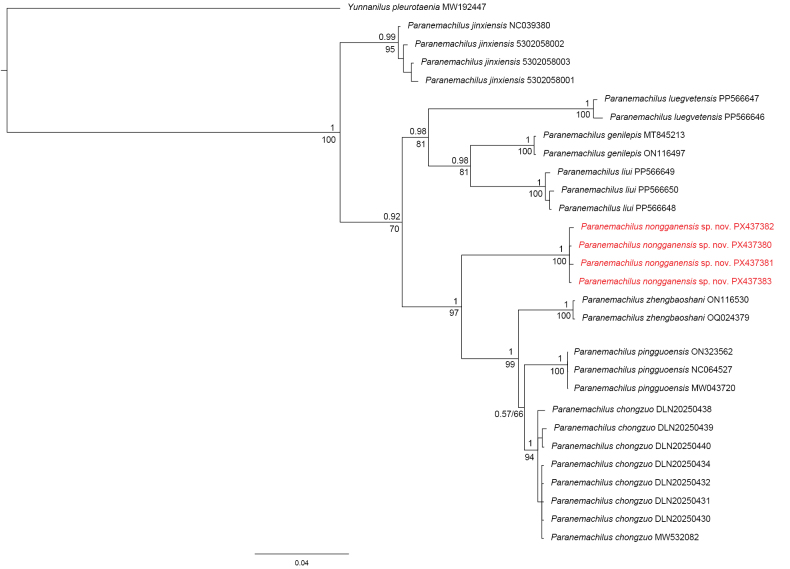
Bayesian phylogram of *Paranemachilus* based on mitochondrial Cyt *b* sequences. Numbers on branches represent BPPs from BI and bootstrap supports from ML.

The BI and ML analyses produced fully congruent topologies. The BI tree is presented here with ML bootstrap values annotated at the nodes. BI and ML analyses of the phylogenetic tree yielded a well-resolved and consistent topology and confirmed the validity of the new species with high nodal support (posterior probability = 1; bootstrap support = 100). Species of *Paranemachilus* were resolved as a monophyletic group, positioned as the sister lineage to the clade containing *Troglonectes*. Within the genus, *P. nonggangensis* sp. nov. was resolved as the sister taxon to the clade containing *P. pingguoensis*, *P. chongzuo*, and *P. zhengbaoshani* (Fig. 1).

The uncorrected *p*-distance analysis based on Cyt *b* sequences further supported species-level distinction, with genetic divergences between *P. nonggangensis* sp. nov. and the nine congeners ranging from 5.18% (for *P. chongzuo*) to 7.67% (for *P. luegvetensis*) (Table 1).

**Table 1.** Uncorrected *p*-distances (%) between species of *Paranemachilus* based on mitochondrial Cyt *b* gene.

ID	Species	1	2	3	4	5	6	7
1	*P. nonggangensis* sp. nov.							
2	*P. chongzuo*	5.18						
3	*P. genilepis*	7.15	6.49					
4	*P. jinxiensis*	7.03	6.59	5.78				
5	*P. liui*	7.45	6.16	3.92	6.57			
6	*P. luegvetensis*	7.67	8.21	7.00	7.47	7.12		
7	*P. pingguoensis*	6.06	2.03	7.17	6.82	7.07	8.91	
8	*P. zhengbaoshani*	5.95	2.55	7.63	7.05	6.95	8.44	3.39

#### Paranemachilus nonggangensis sp. nov.

Taxon classificationAnimaliaCypriniformesNemacheilidae

5E4AEB06-FBF0-5603-9459-B4A0EBC0C465

https://zoobank.org/605B3322-760E-4E9C-94CD-64B3C5F1175C

##### Type material.

***Holotype*** • KIZ2024010571, 70.4 mm SL, Administration Center of Guangxi Nonggang National Nature Reserve, Chongzuo City situated within the Zuojiang River system in Guangxi Zhuang Autonomous Region, China, 22.4325°N, 106.9098°E, elevation 177 m, Z.-Q. Nong and J.-J. Zhou, collected in October 2024.

***Paratypes*** • KIZ2024010572–10573, 10626–10632, nine specimens, 65.9–94.1 mm SL, same data as holotype.

***Molecular materials*** • GXNU-GJY2405052601–4, same data as holotype.

***Bone scan materials*** • GXNU- DLN20240595, same data as holotype.

##### Diagnosis.

The new species is assigned to the genus *Paranemachilus* based on a phylogenetic analysis and morphological traits. Morphologically, *P. nonggangensis* sp. nov. can be distinguished from all other species of the genus *Paranemachilus* by the following combination characters: cheek scaled (vs scaleless in *P. chongzuo*, *P. pingguoensis*, and *P. zhengbaoshani*), 12 or 13 branched pectoral fin rays (vs 13 or 14 in *P. jinxiensis*, 10 *in P. chongzuo*), 6–10 supraorbital canal pores (>11 in *P. genilepis*, *P. luegvetensis*, and *P. liui*), outer gill raker present on first gill arch (vs absent in *P. jinxiensis* and *P. genilepis*), 3+11–15 infraorbital canal pores (vs <10 in *P. zhengbaoshani*, *P. chongzuo*, and *P. jinxiensis*; 7+18–19 in *P. luegvetensis*).

##### Description.

The morphometric data for *P. nonggangensis* sp. nov. are provided in Table 2. Dorsal fins with three unbranched and eight branched rays; pectoral fin with one unbranched and 12 or 13 branched rays; pelvic fin with one unbranched and six branched rays; anal fin with two unbranched and five branched rays; caudal fin with two unbranched and 17 branched rays.

**Table 2.** Morphometric meristic data of *Paranemachilus nonggangensis* sp. nov.

Characters	Holotype	Paratypes (*N* = 9)	Mean ± SD
Total length (mm)	84.7	79.0–112.3	85.6 ± 10.5
Standard length (mm)	70.4	65.9–94.1	71.2 ± 8.9
Percentage of standard length (%)			
Body depth	19.5	18.4–22.4	19.8 ± 1.3
Head width	15.1	14.2–16.6	15.2 ± 0.8
Head length	24.5	22.7–26.1	25.0 ± 1.1
Predorsal length	56.8	55.9–59.9	57.7 ± 1.3
Prepelvic length	56.9	55.9–62.2	59.4 ± 1.9
Preanal length	80.3	78.8–84.0	80.6 ± 1.6
Preanus length	76.9	75.5–80.2	77.6 ± 1.5
Pectoral fin length	19.5	16.8–20.6	19.3 ± 1.2
Pelvic fin length	14.1	12.9–15.2	14.0 ± 0.7
Caudal peduncle length	11.4	9.3–12.2	11.0 ± 1.1
Caudal peduncle depth	13.1	11.8–14.0	13.0 ± 0.7
Percentage of head length (%)			
Head depth	56.7	40.1–59.8	55.2 ± 3.3
Snout length	33.6	30.9–36.0	33.4 ± 1.4
Postorbital length	46.3	42.7–53.0	47.8 ± 3.4
Eye diameter	18.8	10.5–21.1	17.6 ± 3.8
Interorbital width	25.0	25.3–39.2	30.2 ± 4.8
Percentage of caudal peduncle depth (%)			
Caudal peduncle length	87.3	69.3–94.3	84.3 ± 7.4
Dorsal-fin rays	iii, 8	iii, 8	
Pectoral-fin rays	i, 13	i, 12–13	
Pelvic-fin rays	i, 6	i, 6	
Anal-fin rays	ii, 5	ii, 5	
Caudal-fin rays	i, 17, i	i, 17, i	

Body robust, slightly compressed, the deepest body depth at anterior dorsal-fin origin, 18.4–22.4% of SL. Head slightly depressed, maximum head width slightly greater than its height. Anterior and posterior nostrils adjacent, base of anterior nostril tube-shaped, with tip elongated, barbel-like; barbel length shorter than half of tube depth. Eyes normal; eye diameter 10.5–21.1% of lateral head length. Snout obtuse, mouth inferior, lips with shallow folds. Three pairs of barbels; inner rostral barbel and outer rostral reaching anterior and posterior margin of eye, respectively; maxillary barbel exceeding posterior margin of opercula.

**Figure 2.**
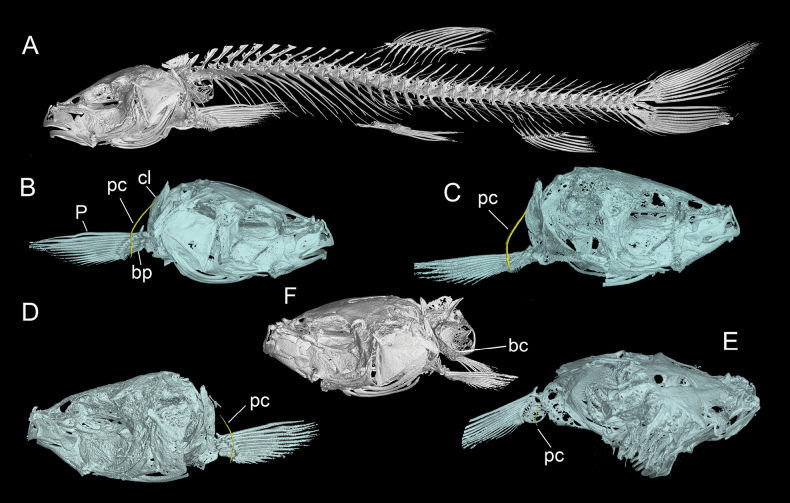
Skeleton of *Paranemachilus* species. **A, B**. *P. nonggangensis* sp. nov.; **C**. *P. genilepis*; **D**. *P. pingguoensis*; **E**. *P. zhengbaoshani*; **F**. *P. chongzuo*. Abbreviations: bc, bony capsule; bp, basipterygium; cl, clavicle; P, pectoral fin; pc, postcleithrum.

**Figure 3.**
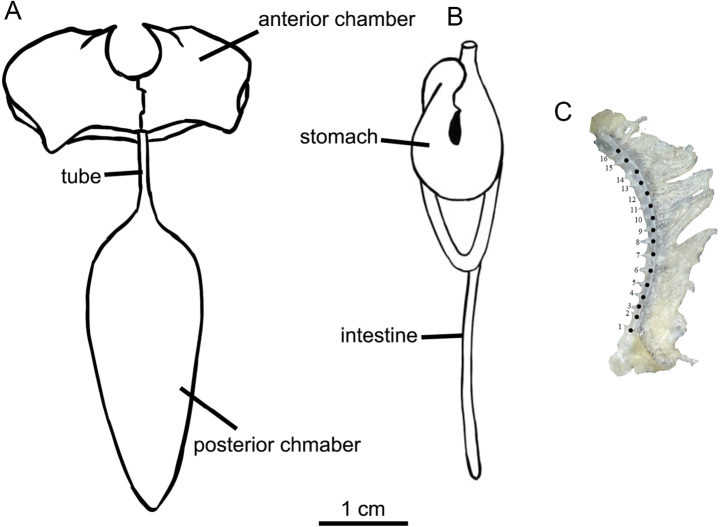
Morphological characteristics of *Paranemachilus nonggangensis* sp. nov. **A**. Anterior and posterior chambers; **B**. Stomach and intestine; **C**. Gill raker.

Dorsal-fin origin is slightly anterior to pelvic-fin origin. Predorsal length 55.9–59.9% of SL; pre-pelvic length 55.8–62.2% of SL; pre-anal length 78.8–84.0% of SL; pre-anus length 75.5–80.2% of SL. Tip of pectoral fin reaching the midpoint between origin of pectoral fin and the origin of pelvic fin. Pelvic-fin origin located vertically below the base of the first branched dorsal fin ray. Tip of pelvic fin not reaching anus. Anus close to the anal fin origin. Caudal fin emarginated; caudal peduncle with adipose crests along both dorsal and ventral sides. Caudal peduncle length 69.3–94.3% of its depth. Cephalic lateral line system well developed, with 3+11–15 infraorbital canal pores, 6–10 supraorbital canal pores, 4–6 supratemporal canal pores, 6–12 preoperculomandibular canal pores. Lateral line incomplete, with 18–22 pores, disappeared behind the vertical at the end of pectoral fin. One outer gill raker and 15–17 inner gill rakers on first gill arch (in three specimens) (Fig. 3C). Vertebrae 3+36, postcleithrum presence (Fig. 2A).

Stomach U-shaped, and intestine has a loop after stomach (Fig. 3A). Two air-bladder chambers: anterior chamber enclosed in a bony capsule which is membranous posteriorly, and developed posterior chamber free in the abdominal cavity; anterior and posterior chambers connected by thin tubes (Fig. 3B).

##### Colouration.

**Figure 4.**
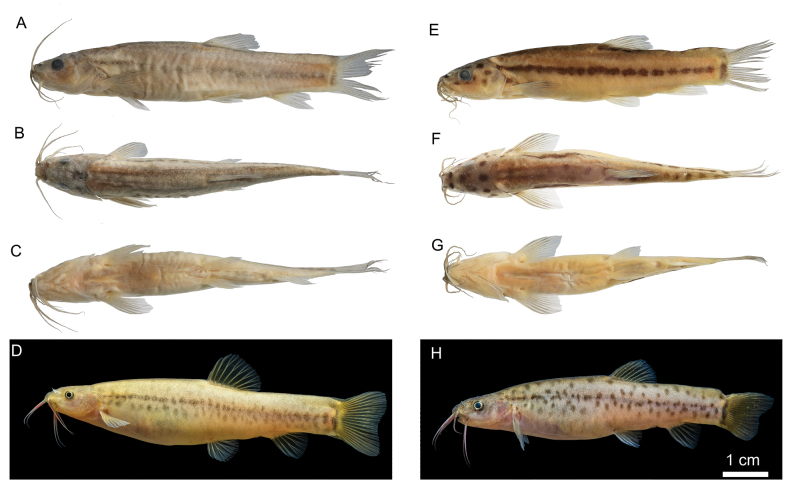
Lateral, dorsal, ventral and living views of *Paranemachilus nonggangensis* sp. nov. **A–D**. Female, holotype KIZ2024010571 (♀); **E–G**. Male, KIZ2024010628; **H**. Male, KIZ2024010573.

In live specimens, the body colour is pale golden yellow. Females have a row of brown maculae along the midline of the body, with a stripe composed of spots beneath these maculae. No obvious spots are present on other parts of the body and head (Fig. 4D). The upper two-thirds of the male body is covered with brown maculae; cheek with two or three unclearly brownish spot, head dorsum has seven brownish sports (Fig. 4H).

In formalin-preserved specimens, the body colour turns pale grey. Females exhibit an indistinct, light-brown stripe along the lateral line, with no obvious spots on other body parts (Fig. 4A–C). Males have a distinct row of dark-brown stripes along the body midline, with other spots on the body sides indistinct. There are four dark-brown spots along the infraorbital pores on the head, two circular spots on the operculum, and seven or eight spots on the dorsal surface of the head. The body dorsum anterior to dorsal fin origin has 7–9 dark-brown spots, and the posterior to dorsal fin base has nine or 10 dark-brown spots (Fig. 4E–G). Fins are hyaline.

##### Distribution and habitat.

**Figure 5.**
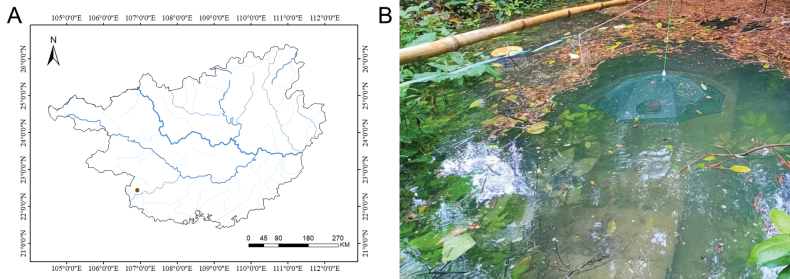
**A**. Distribution of *Paranemachilus nonggangensis* sp. nov. in Guangxi, China; **B**. Habitat photograph of the type locality.

*Paranemachilus nonggangensis* sp. nov. was collected from Administration Center of Guangxi Nonggang National Nature Reserve, Chongzuo City, which is situated within the Zuojiang river system in Guangxi Zhuang Autonomous Region, China (Fig. 5A). This species mainly inhabits seasonal ponds formed during the rainy season or underground rivers. The ponds dry up completely in the dry season, prompting the species to move into underground karst caves. The substrate of its habitat consists of large rocks, with a water depth ranging from approximately 1–3 m. Hydrophilous plants grow in the surrounding area, and there is a considerable amount of dead branches and fallen leaves which float on the water surface (Fig. 5B).

##### Etymology.

The specific epithet refers to Nonggang National Nature Reserve, the type locality. We suggest the Chinese common name “弄岗异条鳅” (NÒng Găng Yì Tiáo Qiū).

## Discussion

In karst landforms, small, fragmented water bodies, such as isolated cave lakes and segmented underground river channels, form natural barriers that block gene flow among species in different water bodies. This is one of the core conditions triggering allopatric speciation, a process that is particularly common in the differentiation of karst-endemic fish (Luo et al. 2024; Mo et al. 2024; Chen et al. 2025). With the development of molecular biology, fish taxonomy has shifted from traditional morphological classification to an approach that combines morphological data with molecular results, leading to the description and publication of an increasing number of cryptic species (e.g. Du et al. 2021, 2023; Lan et al. 2023; Shao et al. 2025).

Previously, *Paranemachilus genilepis* was thought to have a wide distribution in Guangxi. However, with the successive description of new species such as *P. chongzuo*, *P. luegvetensis*, and *P. liui*, the genus *Paranemachilus* may actually be a relatively specialised taxon. Although these species are quite similar in morphology, they exhibit significant genetic distances at the molecular level. The distinct genetic divergence of *P. nonggangensis* sp. nov., as evidenced by an uncorrected *p*-distance of 5.18% from *P. chongzuo*, along with clear morphological differentiation, proves the validity of this new species from a taxonomic perspective.

Five species of *Paranemachilus*, *P. chongzuo*, *P. genilepis*, *P. nonggangensis* sp. nov., *P. pingguoensis*, and *P. zhengbaoshani* were scanned, the final CT reconstructed skull images confirmed the presence of the postclethrum in most species except *P. chongzuo* (Fig. 2B–F). Additionally, the cheeks of *P. genilepis* and *P. nonggangensis* are scaled, while in *P. pingguoensis* and *P. zhengbaoshani* the cheeks are scaleless. Although the presence or absence of cheek scales and the postclethrum are important diagnostic characters for the genera *Heminoemacheilus* and *Paranemachilus*, molecular results do not support the taxonomic independence of *Heminoemacheilus*. Therefore, in this study, we support the view of Bănărescu and Nalbant (1995) that the presence or absence of cheek scales and the postcleithrum are interspecific differences. Consequently, the diagnosis of the genus *Paranemachilus* is characterised by the following key features: anterior and posterior nostril adjacent (separated by a distance shorter than the diameter of the posterior nostril), anterior nostril with a tubular base and its tip elongated into a barbel-like projection, which is shorter than half the depth of the nostril tube; lateral line incomplete; entire body covered with minute scales; bony capsule of the open-type; posterior chamber of swim bladder well developed; caudal fin forked or emarginated.

Morphologically, *P. nonggangensis* sp. nov. can be further distinguished from congeneric species of *Paranemachilus* by having 12 or 13 branched pectoral fin rays (vs 10 in *P. chongzuo*), six branched pelvic fin rays (vs seven or eight in *P. jinxiensis*), outer gill raker on first gill arch present (vs absent in *P. chongzuo*, *P. jinxiensis*, and *P. genilepis*), 11–15+3 infraorbital canal pores (vs <10+3 in *P. chongzuo*, *P. jinxiensis*, and *P. zhengbaoshani*, 18–19+7 in *P. luegvetensis*), caudal peduncle length 9.3–12.2% of SL (vs 12.5–17.8% in *P. liui*).

## Supplementary Material

XML Treatment for Paranemachilus nonggangensis

## Additional information

### Use of AI

No use of AI was reported.

### Author ORCIDs

Jia-Jun Zhou https://orcid.org/0000-0003-1038-1540

Li-Na Du https://orcid.org/0000-0002-2246-643X

### Data availability

All of the data that support the findings of this study are available in the main text.
